# Predicting assembly mode of membraneless organelles by a FRET-based crowding sensor

**DOI:** 10.1038/s41392-023-01435-2

**Published:** 2023-06-12

**Authors:** Feng Chen, Siyuan Shen, Xu Cao, Liang Zhang, Lunxu Liu, Daoke Yang, Yunyu Shi, Wei He, Xuebiao Yao, Dan Liu

**Affiliations:** 1 MOE Key Laboratory for Membraneless Organelles and Cellular Dynamics, Hefei National Research Center for Physical Sciences at the Microscale, Division of Life Sciences and Medicine, University of Science and Technology of China, Hefei, 230027 China; 2 Department of Thoracic Surgery/Institute of Thoracic Oncology, West China Hospital, Sichuan University, Chengdu, 610065 China; 3 Cancer Hospital of the First Affiliated Hospital of Zhengzhou University, Zhengzhou, 450052 China

**Keywords:** Cell biology, Molecular biology

**Dear Editor**,

Biomacromolecules aggregate to form membraneless organelles (MLOs) via phase separation has been observed in a variety of physiological processes over the last decade.^1^ However, the molecular basis of biomacromolecule phase separation in cells and assembly modes of this process remain largely unknown due to a lack of available experimental methods. The dynamics of different MLOs vary widely. For example, in the nucleus of eukaryotic cells, nuclear speckles have been shown to form membraneless organelles by phase separation, and it is mainly involved in the highly dynamic biological process of pre-mRNA splicing.^2^ In contrast, the structure of heterochromatin regions is relatively stable.^3^ In this study, we investigated whether heterochromatin protein HP1α and nuclear speckle protein SRSF2, two classical phase separation proteins with different dynamics, share the same basic unit and similar assembly mode during the formation of MLOs.

At the outset of the study, we compared the mobility of heterochromatin protein HP1α and nuclear speckle protein SRSF2 using fluorescence recovery after photobleaching (FRAP) assay. It has been shown that the mobility of SRSF2 is indeed much higher than that of HP1α (Fig. 1a, b). Next, mEGFP-SRSF2 and mEGFP-HP1α proteins were expressed in *Escherichia coli* (*E. coli*). During in vitro purification, mEGFP-SRSF2 was found to be eluted into the external water from the void volume of the molecular sieve, while the mEGFP-HP1α signal appeared at a later stage (Supplementary Fig. S1a–c). Subsequent static light scattering experiments revealed that mEGFP-HP1α was mainly present in the form of a dimer, with a molecular weight of approximately 114 kDa (Fig. 1c). In contrast, mEGFP-SRSF2 was predominantly present in the form of a large polymer with a total molecular weight of approximately 2.9 × 10^5^ kDa, indicating that approximately 5000 mEGFP-SRSF2 molecules were present in each unit of polymer (Fig. 1d). Furthermore, native-PAGE analysis confirmed the existence form of SRSF2 and HP1α in the natural state (Supplementary Fig. S2). The results of fluorescence microscopy demonstrated that no droplets formed due to the aggregation of the two purified proteins prior to the addition of PEG8000. We then added 10% PEG8000 to each purified protein system and observed successful phase separation in vitro (Fig. 1e). We also explored the effect of nucleic acids on the mobility of these two proteins by in vitro FRAP assay (Supplementary Fig. S3a, b). These results indicated that HP1α and SRSF2 were already present as dimers and large polymers, respectively, prior to phase separation. Therefore, we investigated the forms of the two proteins in mammalian cells. We assessed whether the two proteins behaved similarly in cells by processing HEK293T cells by ultrasonication, passing the cell lysates through a molecular sieve, and collecting the cellular components at different migration speeds. Localizations of SRSF2 and HP1α were measured by Western blot analysis. The cellular experimental results were consistent with the in vitro findings. Endogenous SRSF2 protein in the cell lysate mainly appeared in the external water, while approximately 95% of HP1α protein could enter the molecular sieve (Fig. 1f, g).

**Fig. 1:**
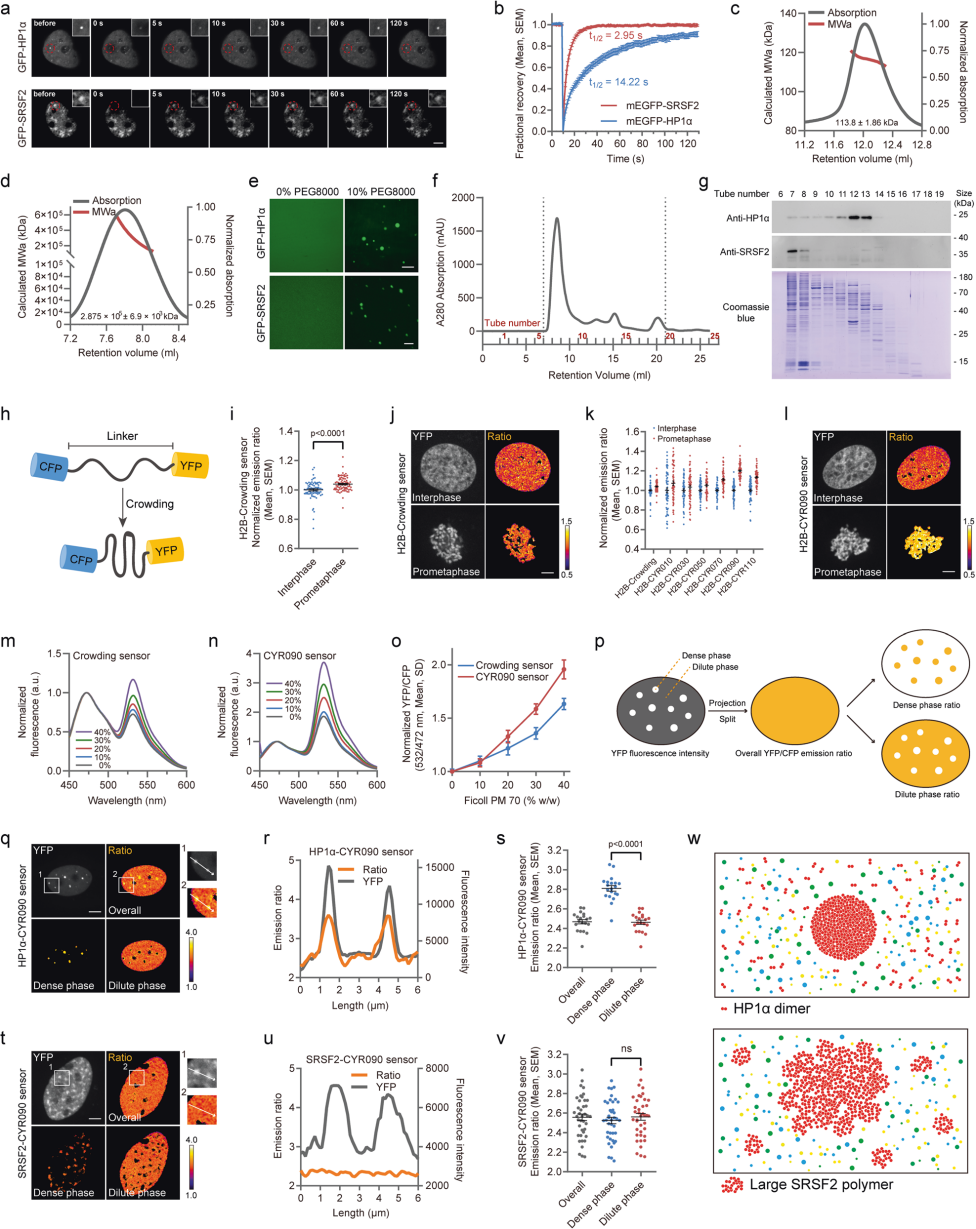
Predicting assembly mode of membraneless organelles by a FRET-based crowding sensor. **a** Representative images of mEGFP-HP1α and mEGFP-SRSF2 from the FRAP experiment. Scale bar, 5 μm. **b** Quantification of FRAP data for mEGFP-HP1α and mEGFP-SRSF2. The bleaching event occurred at *t* = 10 s. *N* = 12 cells per group. Data are represented as mean ± SEM. **c**, **d** SEC-MALS analysis of the purified mEGFP-HP1α protein (**c**) and mEGFP-SRSF2 protein (**d**). **e** Phase separation assay of 10 μM purified mEGFP-HP1α and mEGFP-SRSF2 protein in 10% PEG8000 (w/v) in vitro. Scale bar, 10 μm for HP1α and 2 μm for mEGFP-SRSF2. **f** Separation of HEK293T cell lysates by gel-filtration chromatography. Products were collected separately in 1.5-ml tubes from 6 ml to 19 ml. **g** SDS-PAGE analysis of separated HEK293T cell lysates stained with Coomassie brilliant blue and Western blot analysis of HP1α and SRSF2. **h** Schematic diagram of the structure and working principle of the sensor used to detect the degree of crowding. **i** Comparison of the YFP/CFP emission ratios of the H2B-fused Crowding sensor between cells at interphase and prometaphase. *N* = 80 cells per group from four independent experiments. Data are represented as mean ± SEM. **j** A color-coded representation of the emission ratio in (**i**). Scale bar, 5 μm. **k** Comparison of the YFP/CFP emission ratio of the H2B-fused Crowding sensor and optimized sensor to detect the degree of crowding containing random amino acid sequences of different lengths. *N* ≥ 60 cells per group from four independent experiments. Data are represented as mean ± SEM. **l** A color-coded representation of the emission ratio of the H2B-fused CYR090 sensor in (**k**). Scale bar, 5 μm. **m**, **n** Fluorescence emission spectra upon titration of the prokaryotically expressed and purified Crowding sensor (**m**) and CYR090 sensor (**n**) with Ficoll PM 70. The fluorescence emission spectra in (**m**) and (**n**) were normalized according to their donor fluorescence. **o** FRET ratio upon titration with Ficoll PM 70. *N* = 3 independent experiments. Data are represented as mean ± SD. The emission ratios in (**i**), (**j**), (**k**), (**l**) and (**o**) were normalized according to the corresponding control group. **p** Schematic diagram of YFP/CFP emission ratio image splitting method for dense phase and dilute phase. **q** Comparison of the degree of HP1α crowding in heterochromatin (dense phase) and nucleoplasm (dilute phase). A representative image of HP1α-CYR090 sensor fluorescence intensity (YFP) and corresponding color-coded image of the overall and separate YFP/CFP emission ratio. Scale bar, 5 μm. Higher ratio indicates higher degree of crowding. **r** Two curves in different colors showing relative trends in YFP fluorescence intensity (gray) and YFP/CFP emission ratio (orange) in (**q**). **s** Comparison of YFP/CFP emission ratio of HP1α-CYR090 sensor in different regions. *N* = 20 cells per group. Data are represented as mean ± SEM. **t**, **u** Comparison of the degree of SRSF2 crowding in nuclear speckles (dense phase) and the nucleoplasm (dilute phase). The images in (**t**) and (**u**) are similar to those in (**q**) and (**r**), except that the SRSF2-CYR090 sensor was used. **v** Comparison of YFP/CFP emission ratio of SRSF2-CYR090 sensor in different regions. *N* = 40 cells per group from two independent experiments. Data are represented as mean ± SEM. **w** Oligomeric molecule-mediated aggregation model (top), and polymeric molecule-mediated consolidation model (bottom)

We then attempted to explore the reasons for different behaviors of the two proteins by probing changes in crowding degree. Bert Poolman and colleagues previously reported a fluorescence resonance energy transfer (FRET)-based Crowding sensor for quantification of macromolecular crowding in living cells (Fig. 1h).^4^ To verify that the Crowding sensor was representative of the crowding degree change in the environment in which a specific target protein is located, we fused the Crowding sensor to the C-terminus of histone H2B and measured its sensitivity during chromosome condensation. Our results confirmed that the degree of crowding around H2B increased after entering mitosis (Fig. 1i). However, the amplitude of this increase was not large enough for it to be clear on the FRET ratio image (Fig. 1j), which may be due to insufficient sensor sensitivity. Previous study reported that the length of the linker in the sensor used to detect the degree of crowding greatly affects the sensitivity of the sensor.^5^ Thus, we replaced the linker of the Crowding sensor with random amino acid sequences of different lengths (Supplementary Fig. S4a). We then fused the sensors to H2B, transfected them into HeLa cells, and confirmed their expression by Western blot analysis (Supplementary Fig. S4b). A comparison of the FRET ratios of cells at interphase and mitosis revealed that the sensor with a 90-amino acids linker showed the best response (Fig. 1k, l), which we then named the “CYR090 sensor” and used in subsequent experiments. We also performed a series of experiments to verify the specificity of the CYR090 sensor (Supplementary Fig. S4c–h). The sensitivity of the optimized CYR090 sensor and the previously reported Crowding sensor were further compared by purifying these two sensors in vitro and analyzing their fluorescence using a fluorescence spectrophotometer (Fig. 1m–o).

Our results demonstrated that the CYR090 sensor may be an effective tool for detecting the degree of crowding around a specific location in cells. HP1α and SRSF2 were fused to the N-terminus of the CYR090 sensor and transiently expressed in HeLa cells to track the crowding degree around both proteins during phase separation to measure their agglutination characteristics. At the beginning of the experiment, we first excluded the effect of CYR090 sensor on the function and mobility of the fusion proteins (Supplementary Fig. S5 and S6a, b). Cellular YFP fluorescence intensity images were used to determine the precise area in which phase separation occurred. The area with the highest YFP fluorescence intensity was referred to as the “dense phase” and the area with lowest YFP fluorescence intensity was referred to as the “dilute phase.” We then projected the areas on the FRET ratio image to split the image into dense and dilute phase regions (Fig. 1p). These regions were compared and the typical characteristics of HP1α during phase separation were consistent with our assumption. The dense phase, which exhibited higher fluorescence intensity, was also more crowded (Fig. 1q–s). Moreover, consistent with HP1α-CYR090 sensor, CYR090-HP1α, in which sensor was fused to the N-terminus of HP1α, also exhibited significantly higher crowding degree in dense phase than dilute phase (Supplementary Fig. S7a, b). However, highly polymerized SRSF2 showed completely different characteristics. The area with the higher fluorescence intensity did not represent the area with a higher degree of crowding (Fig. 1t–v). Consistent results were obtained by intermolecular FRET assay (Supplementary Fig. S8a–d). We also confirmed that the difference in crowding characteristics was not caused by the fluorescence intensity by analyzing the fluorescence intensity distribution characteristics of HP1α, SRSF2 and two other phase separation proteins, G3BP1 and FUS (Supplementary Fig. S9a–d and S10a, b). Furthermore, 1,6-Hexanediol was used to explore their intermolecular force characteristics (Supplementary Fig. S11a, b).

Based on our observation, we predict that proteins tend to phase separate exists in at least two forms: oligomers and large polymers, as basic phase separation units, and the modes of agglutination for these two existing forms may differ. We propose two potential assembly models for phase separation: an oligomeric molecule-mediated aggregation model, wherein oligomers aggregate to form phase-separated condensates, and a polymeric molecule-mediated consolidation model, in which existing large polymers join to form larger condensed areas during phase separation (Fig. 1w).

## Supplementary information

Supplementary_Materials

## Data Availability

All the data used for the current study are available from the corresponding author upon reasonable request.
