# Supplementary material for: Predicting assembly mode of membraneless organelles by a FRET-based crowding sensor
Source: Signal Transduct Target Ther. 2023 Jun 12;8:227. doi: 10.1038/s41392-023-01435-2 (PMC10258192; doi:10.1038/s41392-023-01435-2)
Supplement: Supplementary file 1 — Supplementary_Materials [file 41392_2023_1435_MOESM1_ESM.docx]

Supplementary Materials for

Predicting assembly mode of membraneless organelles by a FRET based crowding sensor

Feng Chen^1,#^, Siyuan Shen^1,#^, Xu Cao^1^, Liang Zhang^1^, Lunxu Liu^2^, Daoke Yang^3^, Yunyu Shi^1^, Wei He^2,*^, Xuebiao Yao^1,*^, Dan Liu^1,*^

^1^MOE Key Laboratory for Membraneless Organelles and Cellular Dynamics, Hefei National Research Center for Physical Sciences at the Microscale, Division of Life Sciences and Medicine, University of Science and Technology of China; Hefei 230027, China

^2^Department of Thoracic Surgery/Institute of Thoracic Oncology, West China Hospital, Sichuan University; Chengdu 610065, China

^3^Cancer Hospital of the First Affiliated Hospital of Zhengzhou University; Zhengzhou 450052, China

^#^These authors contributed equally: Feng Chen, Siyuan Shen

*Correspondence to: Wei He (hewei@wchscu.cn); Xuebiao Yao (yaoxb@ustc.edu.cn); Dan Liu (dliu919@ustc.edu.cn)

**This PDF file includes:**

Materials and Methods

Extended results

Discussion

Figures S1 to S11

Original and uncropped films of Western blots

Supplemental references

**Materials and Methods**

Cell culture and transfection

HeLa cells and HEK293T cells were cultured in high-glucose DMEM supplemented with 10% fetal bovine serum and 1% penicillin-streptomycin at 37 °C in a humidified incubator containing 5% CO_2_. Cells were transfected with plasmid DNA using Lipofectamine 3000 (Thermo Fisher Scientific) following the manufacturer’s instructions.

Plasmids and Antibodies

The CYR090 sensor was generated by inserting a random linker sequence of 90 amino acids (AGTYNTSTDADRGKPTQGEKDDSKHMTQIDEENPIMNIGKKDLPSRDRKCGP

GMSAGQGHASTLDSPYQHCICGSMAWGPGQVHDPDSGG) between a fluorescent protein pair for FRET. The CyPet-YPet variants of CFP-YFP, which were optimized for FRET ^1^, were used to maximize the sensitivity and dynamic range of the sensor. To avoid the effect of weak dimerization between fluorescent proteins, we introduced A206K point mutations in CFP and YFP ^2^. Genes expressing different localization proteins were cloned into the N terminus (HP1α-CYR090, SRSF2-CYR090, G3BP1-CYR090) or C terminus (CYR090-HP1α, CYR090-FUS) of the CYR090 sensor expressing gene.

Genes encoding human HP1α and SRSF2 were cloned into the pmEGFP-C3 (Clontech) vector with an N-terminal mEGFP tag (mEGFP-HP1α, mEGFP-SRSF2) or the pcDNA3.1 vector with a C-terminal CFP or YFP tag (HP1α-CFP, HP1α-YFP, SRSF2-CFP, SRSF2-YFP). Genes encoding human SRSF1, HP1β and TRIM28 were cloned into the pcDNA3.1 vector with an N-terminal mCherry tag (mCherry-SRSF1, mCherry-HP1β, mCherry-TRIM28).

SRSF2 (20371-1-AP, 1:1000 dilution for WB), GFP (50430-2-AP, 1:2000 dilution for WB), GAPDH (60004-1-Ig, 1:10000 dilution for WB) antibody was purchased from Proteintech, and HP1α (ab109028, 1:10000 dilution for WB) antibody was purchased from Abcam. SON (sc-398508, 1:100 dilution for IF) antibody was purchased from Santa Cruz Biotechnology.

Protein expression and purification

The full-length protein coding sequences of human SRSF2 and HP1α were cloned into a modified pET28a (Novagen) vector containing the mEGFP protein fused at the N-terminus following the 6 × His tag and the 14-amino acid linker sequence GAPGSAGSSSGGSG. The fusion clones of the Crowding sensor and CYR090 sensor were amplified by overlap extension PCR and cloned into the pET22b vector at the C-terminus following the 6 × His tag.

All proteins except mEGFP-SRSF2 were expressed in E. coli BL21 (DE3) cells. Cells were grown in Luria-Bertani medium at 37 °C until the OD600 reached approximately 0.8–1.0. Protein expression was induced with 0.5 mM β-D-1-thiogalactopyranoside (IPTG) for 24 h at 16 °C, after which the cells were collected by centrifugation.

mEGFP-HP1α, the Crowding sensor and the CYR090 sensor were purified as follows. Cell pellet from 1 L of culture was suspended in 50 ml of pre-cooled buffer A (50 mM Tris-HCl, pH 7.5, 500 mM NaCl) and lysed by sonication. The crude lysate was then centrifuged at 13000 rpm for 30 min at 4 °C. The supernatant was subjected to affinity chromatography using a Ni^2+^-chelating resin (GE Healthcare) and then eluted with pre-cooled buffer B (20 mM Tris-HCl, pH 7.5, 500 mM NaCl, 500 mM imidazole). The proteins were further purified by size-exclusion chromatography on a HiLoad 16/60 Superdex 200 column (GE Healthcare) in buffer C (20 mM Tris-HCl, pH 7.5, 500 mM NaCl, 1 mM DTT). HP1α was dialyzed against two changes of buffer D (50 mM Tris-HCl, pH 7.5, 125 mM NaCl, 1 mM DTT, 10% glycerol) at 4 °C. Crowding sensor and CYR090 sensor were dialyzed against buffer E (20 mM Tris-HCl, pH 7.5, 300 mM NaCl, 2.5 mM EDTA) at 4 °C.

mEGFP-SRSF2 was purified as previously described ^3^ with some modifications. mEGFP-SRSF2 was expressed in E. coli BL21 (DE3) cells grown in Luria-Bertani medium at 37 °C until the OD600 reached approximately 0.8–1.0. Protein expression was induced with 1 mM β-D-1-thiogalactopyranoside (IPTG) for 5 h at 37 °C, after which the cells were collected by centrifugation. Cells paste from 1 L of culture was suspended in 50 ml of pre-cooled buffer F (50 mM Tris-HCl, pH 7.5, 500 mM NaCl, 1 mM DTT, 8 M urea) with complete protease inhibitors (Roche, 11873580001) and sonicated (ten cycles of 15 seconds on, 60 seconds off). The lysates were cleared by centrifugation at 13000 rpm for 30 min, and the protein-containing supernatant was subjected to affinity chromatography using a Ni^2+^-chelating resin (GE Healthcare) eluted with pre-cooled buffer G (50 mM Tris-HCl, pH 7.5, 500 mM NaCl, 1 mM DTT, 8 M urea, 500 mM imidazole) with complete protease inhibitors (Roche, 11873580001). Then, the proteins were dialyzed first against a buffer containing 50 mM Tris-HCl, pH 7.5, 500 mM NaCl, 1 mM DTT and 4 M urea, then with the same buffer containing 2 M urea and finally 2 changes of buffer containing 10% glycerol and no urea. Any precipitate after dialysis was removed by centrifugation at 3,000 rpm for 10 min. The proteins were further purified by size-exclusion chromatography on a HiLoad 16/60 Superdex 200 column (GE Healthcare) in buffer H (50 mM Tris-HCl, pH 7.5, 500 mM NaCl, 1 mM DTT, 10% glycerol). The protein was dialyzed against two changes of buffer D at 4 °C.

Size exclusion multi-angle light scattering (SEC-MALS)

Two hundred microlitres of the mEGFP-SRSF2 and HP1α proteins at 1 mg/ml was injected into a Superdex 200 Increase 10/300 GL column at a flow rate of 0.5 ml/min in buffer I (50 mM Tris-HCl, pH 7.5, 500 mM NaCl, 1 mM DTT) using an ÄKTA purifier. The system was coupled on-line to an 8-angle MALS detector (DAWN HELEOS II, Wyatt Technology) and a differential refractometer (Optilab T-rEX, Wyatt Technology). Data were analysed using ASTRA 7.0.1.24.

*In vitro* phase separation assay

Recombinant mEGFP-SRSF2 and HP1α were concentrated and desalted to an appropriate protein concentration with 125 mM NaCl using Amicon Ultra centrifugal filters (10 KMWCO, Millipore). The recombinant proteins were added to droplet formation buffer (50 mM Tris-HCl, pH 7.5, 125 mM NaCl, 1 mM DTT, 10% glycerol) with the indicated amount of crowing agent (PEG8000).

Preparation of nuclear extracts (NE) and total RNA

Nuclear extracts were prepared using a Nucleosome Preparation Kit (Active Motif) according to the manual. We obtain oligo-nucleosomes rather than mono-nucleosomes by controlling the digestion time of Micrococcal Nuclease. Oligo-nucleosomes thus prepared were dialyzed against droplet formation buffer mentioned above. The concentrations of nuclear extracts were expressed as DNA concentrations calculated from the absorbance at 260 nm.

Total RNA was prepared using a Total RNA Extraction Kit (Promega) according to the manual.

*In vitro* characterization of the sensors

These experiments were performed as previously described ^4^ with some modifications. The purified Crowding sensor and CYR090 sensor in buffer E were placed in a quartz cuvette, and their fluorescence emission spectrum after excitation at 435 nm was recorded at 25 °C on a Fluorolog-2700 (HITACHI) spectrofluorometer. To further compare the sensitivity of the optimized CYR090 sensor and Crowding sensor, they were mixed with crowding agent (sucrose, Ficoll PM 70 or Ficoll PM 400) at different concentrations, and the fluorescence emission spectrum was then recorded after excitation at 435 nm. Each experiment was repeated at least three times.

To further identify the influence of the increased intermolecular FRET efficiency caused by aggregation on the FRET ratio, we used proteinase K to digest the linker region of the CYR090 sensor. After digestion with proteinase K and enzymatic hydrolysis of the protein, the protein was identified by SDS-PAGE. Next, the CYR090 sensor with and without proteinase K digestion was placed in a quartz cuvette, and the fluorescence emission spectrum after excitation at 435 nm or 515 nm was recorded. Each experiment was repeated at least three times.

Live-cell imaging and data processing

HeLa cells were cultured on 18 mm × 18 mm glass coverslips coated with poly-D-lysine (Sigma-Aldrich) at the bottom of 35-mm dishes (Wuxi NEST Biotechnology Co.,Ltd ). To maintain a suitable environment for cell growth, the coverslips were mounted in custom Rose chambers using L-15 medium without phenol red (Thermo Fisher Scientific), and the temperature was kept at approximately 37°C. Images were obtained with a Nikon Ti confocal microscope with a 100× 1.4 NA objective; an XY Piezo Z stage (Applied Scientific Instrumentation); a spinning disk confocal unit (Yokogawa); an electron multiplier CCD camera (Andor); and an LMM5 laser merge module with 445-nm, 488-nm, and 594-nm lasers (Andor) controlled by IQ3 live-cell imaging software (Andor). Images collected with this system were 16 bit and consisted of 512 × 512 pixels, and the pixel size was 0.12 μm. Image stacks were acquired with 0.5-μm spacing, and 3–5 planes were usually chosen.

For live imaging of differentially targeted CYR090 sensors, CFP was excited at 445 nm, and both CFP and YFP emissions were simultaneously acquired with a beamsplitter (Dual-View, Optical Insights). ImageJ (NIH ImageJ system, Bethesda, MD) software and MATLAB (MathWorks) were used to analyse YFP/CFP emission ratio images as previously described ^5,6^. The emission ratio in a region of interest was calculated by the fluorescence intensity ratio of YFP to CFP after subtraction of the background signal. For SRSF2- and HP1α-targeted sensors, five z planes were acquired with 0.5-μm spacing.

A custom program written in ImageJ was used to split the YFP/CFP emission ratio images into a dense phase region and a dilute phase region. The program judged and recorded the pixel positions of the dense phase and dilute phase in the YFP fluorescence image. The area with stronger YFP fluorescence intensity was defined as the dense phase, and the area with weaker YFP fluorescence intensity was defined as the dilute phase. Then, these pixel positions were projected onto the YFP/CFP emission ratio image, and the emission ratio image was split into two parts accordingly (Fig. 1p).

Cell synchronization

For HeLa cell synchronization, cells were synchronized at the G1/S phase by the addition of 2.5 mM thymidine (Sigma-Aldrich) and released from blockade 20 hours later. To compare cells at interphase and prometaphase, 8 hours after release of the thymidine block, cells were incubated with 100 ng/ml nocodazole for 1 hour so that some cells were blocked in prometaphase.

Fluorescence recovery after photobleaching (FRAP)

For live cells, the FRAP experiment was performed with a Nikon A1R HD25 confocal microscope. mEGFP-SRSF2, mEGFP-HP1α, SRSF2-CYR090 sensor and HP1α-CYR090 sensor were excited by a 488-nm laser, and 40% laser power was used for bleaching. Images were captured every 1 s with a 60× 1.49 NA objective after bleaching.

For *in vitro* assay, the FRAP experiment was performed on a Nikon CSU-W1 SoRa spinning disk confocal microscope with a XY galvo scanning unit. mEGFP-SRSF2 and mEGFP-HP1α were excited by a 488-nm laser, and 5% laser power was used for bleaching. Images were captured every 10 s with a 100× 1.49 NA objective after bleaching.

The fluorescence intensity at the bleached spot was measured with the FIJI plugin FRAP Profiler V2. Fluorescence half-recovery times (t_1/2_) with 95% confidence intervals were extracted from the fitted curves using GraphPad Prism 8.0.1 ^7^.

Native-PAGE

For acidic proteins such as HP1α, the concentrated gel buffer for polyacrylamide gels was 125 mM Tris-HCl, pH 6.8. The separation gel buffer was 375 mM Tris-HCl, pH 8.8. The electrophoresis buffer was 125 mM Tris and 1.25 M glycine.

For basic proteins such as SRSF2, the concentrated gel buffer for polyacrylamide gels was 60 mM KOH-HAc, pH 6.8. The separation gel buffer was 60 mM KOH-HAc, pH 4.3. The electrophoresis buffer was 80 mM β-Ala-HAc, pH 4.4. Note that when performing basic protein electrophoresis, the positive and negative terminals of the power supply need to be reversed.

Immunofluorescence (IF)

HeLa cells were cultured on 18 mm × 18 mm glass coverslips coated with poly-D-lysine (Sigma-Aldrich) at the bottom of 35-mm dishes (Wuxi NEST Biotechnology Co.,Ltd ). After 48 hours of gene transfection the cells were fixed in 4% paraformaldehyde for 10 minutes, followed by permeabilization with PBS buffer containing 0.4% Triton X-100 for 10 minutes. After that, the cells were blocked with PBS buffer containing 5% BSA for 30 min, then incubated sequentially with primary and secondary antibodies.

Statistical analysis

Statistical analyzes for all experiments were performed with the indicated statistical tests using Graphpad Prism software. A two-tailed, unpaired Student’s t-test, was used for experiments with only two groups. P values of 0.05 or less were considered statistically significant.

**Extended results**

Exploring the effect of nucleic acids on the mobility of SRSF2 and HP1α condensates

To compare the difference in mobility of SRSF2 and HP1α condensates *in vitro* as well as exploring the effect of nucleic acids, we expressed mEGFP-SRSF2 and mEGFP-HP1α proteins *in vitro*, and compared the mobility of SRSF2 and HP1α proteins under the four systems of SRSF2, SRSF2 + RNA, HP1α and HP1α + nuclear extracts by FRAP experiments. We found that consistent with the in vivo experiments, SRSF2 was always more mobile than HP1α. SRSF2 added with RNA showed faster recovery and higher mobility, and their t_1/2_ was at the level of several minutes. Comparing with HP1α itself, HP1α added with nuclear extracts showed the worst mobility, and their t_1/2_ was increased from several minutes to several hours. (Supplementary Fig. S3a, b) Thus, we speculate that the higher mobility of SRSF2 than HP1α may be due to its assembly modes rather than RNA or chromatin binding.

Verification of the specificity of the CYR090 sensor

A series of experiments was performed to determine whether the crowding degree of the surrounding environment significantly contributes to the difference in FRET ratio for the CYR090 sensor. There was no significant change in the FRET ratio with the CYR090 sensor when the sensor solution was continuously concentrated by ultrafiltration without the addition of crowding agents, demonstrating that the concentration of the sensor does not influence the FRET ratio (Supplementary Fig. S4c). Second, unlike Ficoll, the addition of different concentrations of sucrose monomer to the sensor solution did not increase the FRET ratio, demonstrating that the change in FRET ratio was not caused by a chemical reaction between the sensor and sucrose (Supplementary Fig. S4d). Third, we further examined whether increased intermolecular FRET efficiency caused by aggregation influences the FRET ratio using proteinase K to digest the linker region in the middle of the sensor (Supplementary Fig. S4e) and observed a significant decrease in the FRET efficiency of the sensor (Supplementary Fig. S4f). The addition of Ficoll PM 70 at different concentrations after enzymolysis did not change the FRET ratio (Supplementary Fig. S4g), which indicated that sensor activity was dominated by the approach of the terminal fluorophores within the molecule toward each other rather than aggregation of the sensors. On the other hand, excitation of the sensor at 515 nm, which is the optimal excitation wavelength for YFP, resulted in almost no change in the fluorescence intensities before and after digestion (Supplementary Fig. S4h), indicating that the change in FRET efficiency was not caused by destruction of the fluorescent protein by proteinase K.

Exploring the effect of CYR090 sensor on the function and dynamics of HP1α and SRSF2

The function of HP1α and SRSF2 is closely related to their localization in cells, so we conducted co-localization experiments in HeLa cells. In addition to SRSF2, SRSF1 and SON are also classical nuclear speckle-related proteins ^8^. We overexpressed mEGFP-SRSF2 or SRSF2-CYR090 together with mCherry-SRSF1 in HeLa cells respectively, and found that CYR090 sensor did not affect the co-localization between SRSF2 and SRSF1. Furthermore, when mEGFP-SRSF2 or SRSF2-CYR090 was overexpressed, immunofluorescence staining was performed on endogenous SON protein in cells, and it was also found that CYR090 sensor had no effect on the co-localization between SRSF2 and SON. To investigate the effect of CYR090 sensor on the function of HP1α protein, we co-transfected mEGFP-HP1α, HP1α-CYR090 or CYR090-HP1α with mCherry-HP1β, or mCherry-TRIM28 respectively, and found that HP1α and CYR090 fusions always colocalize with other heterochromatin related proteins, HP1β and TRIM28 ^9^ (Supplementary Fig. S5). These results indicated that the CYR090 sensor, no matter fused to the N or C terminus of HP1α does not affect the colocalization between HP1α and HP1β or TRIM28. Therefore, we speculate that the CYR090 sensor does not affect the function of HP1α and SRSF2 proteins.

In order to investigate whether CYR090 sensor would affect the mobility of fusion proteins, we overexpressed HP1α-CYR090 and SRSF2-CYR090 respectively in HeLa cells, and performed FRAP assay. The results indicated that not only HP1α-CYR090 and GFP HP1α, but also SRSF2-CYR090 and GFP-SRSF2 have close t_1/2_ (Fig. 1a, b), suggesting that the fusion of CYR090 sensor did not significantly affect the mobility of HP1α and SRSF2 proteins (Supplementary Fig. S6a, b).

Detecting the changes in crowding degree around HP1α and SRSF2 during phase separation by intermolecular FRET assay

In order to confirm the different characteristics of the changes in crowding degree of HP1α and SRSF2 during phase separation, we designed a series of intermolecular FRET experiments. We constructed two sets of plasmids to express HP1α-CFP and HP1α-YFP or SRSF2-CFP and SRSF2-YFP in HeLa cells respectively. The experiment was designed to reflect the changes in crowding degree around different localized proteins through the changes in intermolecular distance. Through the analysis of FRET ratio, we found that the conclusion given by this experiment was consistent with the results of intramolecular sensors. Compared with the dilute phase, the dense phase of HP1α has a higher crowding degree (Supplementary Fig. S8a, b), however, there was no significant difference in crowding between dense phase and dilute phase of SRSF2 (Supplementary Fig. S8c, d).

Detecting the changes in crowding degree around G3BP1 and FUS during phase separation by CYR090 sensor

To test whether other phase separation proteins fit the phase separation assembly models proposed by us, we applied CYR090 sensor to study the phase separation process of G3BP1 and FUS. Among the four proteins studied in the experiment, the intrinsically disordered protein region (IDR) of HP1α accounted for about 25% of the total length of the protein sequence, and the IDR of SRSF2 accounted for nearly 60% (based on UNIPROT database). For G3BP1 and FUS, these numbers are 45% and 80% respectively. The results of FRET assay show that in G3BP1 experiment with relatively low content of IDR, the dense phase has a higher crowding degree than dilute phase which is like HP1 (Supplementary Fig. S9a, b). In FUS experiment with relatively high content of IDR, the crowding degree of dense phase and dilute phase is consistent (Supplementary Fig. S9c, d). This result prompts us to consider whether there are common rules governing the process of phase separation which responds to different molecular events in cells.

The fluorescence intensity ratio of dense phase to dilute phase does not affect the detection of crowding degree

Furthermore, we analyzed the fluorescence intensity ratio of dense phase to dilute phase of HP1α, SRSF2, G3BP1 and FUS when phase separation occurs in cells, and found that G3BP1 and FUS with the highest ratio, but their crowding changes of dense phase and dilute phase are not consistent (Supplementary Fig. S10a, b). Fluorescence intensity ratio of dense phase and dilute phase of HP1α is not as high as FUS, although crowding degree of dense phase of HP1α is higher. These evidences indicate that the crowding changes do not depend on the fluorescence intensity.

Characteristics of intermolecular forces in different phase separation models

1,6-Hexanediol has been shown to disrupt the weak, multivalent, hydrophobic interactions and thereby affect the process of phase separation and has been widely used for *in vitro* and intracellular phase separation studies ^10-12^. To explore the characteristics of the intermolecular forces that drive the formation of phase-separated agglutinates by different assembly modes, we transfected HeLa cells with the HP1α and SRSF2-targeted CYR090 sensor and performed a real-time tracking experiment to measure the crowding degree of these two proteins after the addition of 1,6-hexanediol. The crowding degree around SRSF2 and HP1α both decreased, but from the trend, the crowding degree around SRSF2 decreased more obviously (Supplementary Fig. S11a). As a control, the ratio without the addition of 1,6-hexanediol did not obviously decrease, indicating that the changes in the experimental groups were not caused by fluorescent protein quenching during long-term tracking (Supplementary Fig. S11b). Therefore, we concluded that weak, multivalent, hydrophobic interactions are involved in the formation of SRSF2 condensates, whereas HP1α aggregation may be more dependent on other stronger interaction forces.

**Discussion**

Changes of crowding degree can reflect the basic unit and assembly mode of MLO formation

In the oligomeric molecule-mediated aggregation model, the basic phase separating unit contains only a limited number of molecules. Thus, the crowding degree should increase during phase separation due to the accumulation of a large number of basic units. On the contrary, in the polymeric molecule-mediated consolidation model, since molecules are already highly condensed into large polymer units prior to phase separation, the crowding degree may not increase when these units join. Therefore, the basic unit and assembly mode of MLOs during formation can be explored using a crowding detection biosensor.

Our results in Figures 1q to 1v suggest that many HP1α dimers aggregate during phase separation, which leads to a large increase in the crowding degree within the aggregates. In contrast, SRSF2 exists as a large polymer in the nucleoplasm and the CYR090 sensor mainly indicates the crowding degree within a SRSF2 phase separation unit containing thousands of SRSF2 molecules, which weakens the ability of the CYR090 sensor to detect subtle changes when SRSF2 units consolidate into a larger dense-phase area. Taken together, these results provide direct evidence of the models described in Figure 1w.

Biological significance of MLO formation via different assembly modes

Numerous studies have confirmed that different types of MLOs have different mobility ^13-15^, but the reason for this difference remains unclear. FRAP analysis (Fig. 1a, b) revealed that the mobility of the phase-separated aggregate formed via the aggregation model was significantly weaker than that formed via the consolidation model. Therefore, we speculate that phase-separated condensate formed by the consolidation model may form transient biochemical reaction compartments. Biological macromolecules can assemble these compartments rapidly to deal with the high dynamics of certain biological processes, such as the pre-mRNA alternative splicing process in which SRSF2 is involved ^16^. In contrast, the aggregation model may be applied to some stable reaction processes, such as heterochromatin formation that HP1α participates in. These type of MLOs may require a relatively stable structure formed by the aggregation model instead of the consolidation model which may not be able to meet the requirements in terms of rigidity and stability ^17,18^.

Physiological significance of exploring the basic unit and assembly mode of MLOs

Dysfunction of MLOs has been shown to be associated with various diseases, especially cancer ^19,20^. Exploring the assembly modes of MLOs will help develop treatments for cancers caused by abnormal phase separation processes. The relationship between aberrant pre-mRNA alternative splicing and cancer has gradually become clear ^21^. Splicing regulator SRSF2 has been shown to drive myeloid neoplasms and hepatocellular carcinoma development ^22,23^, and our study on the phase separation characteristics of SRSF2 protein may facilitate the development of anticancer drugs targeting pre-mRNA alternative splicing.


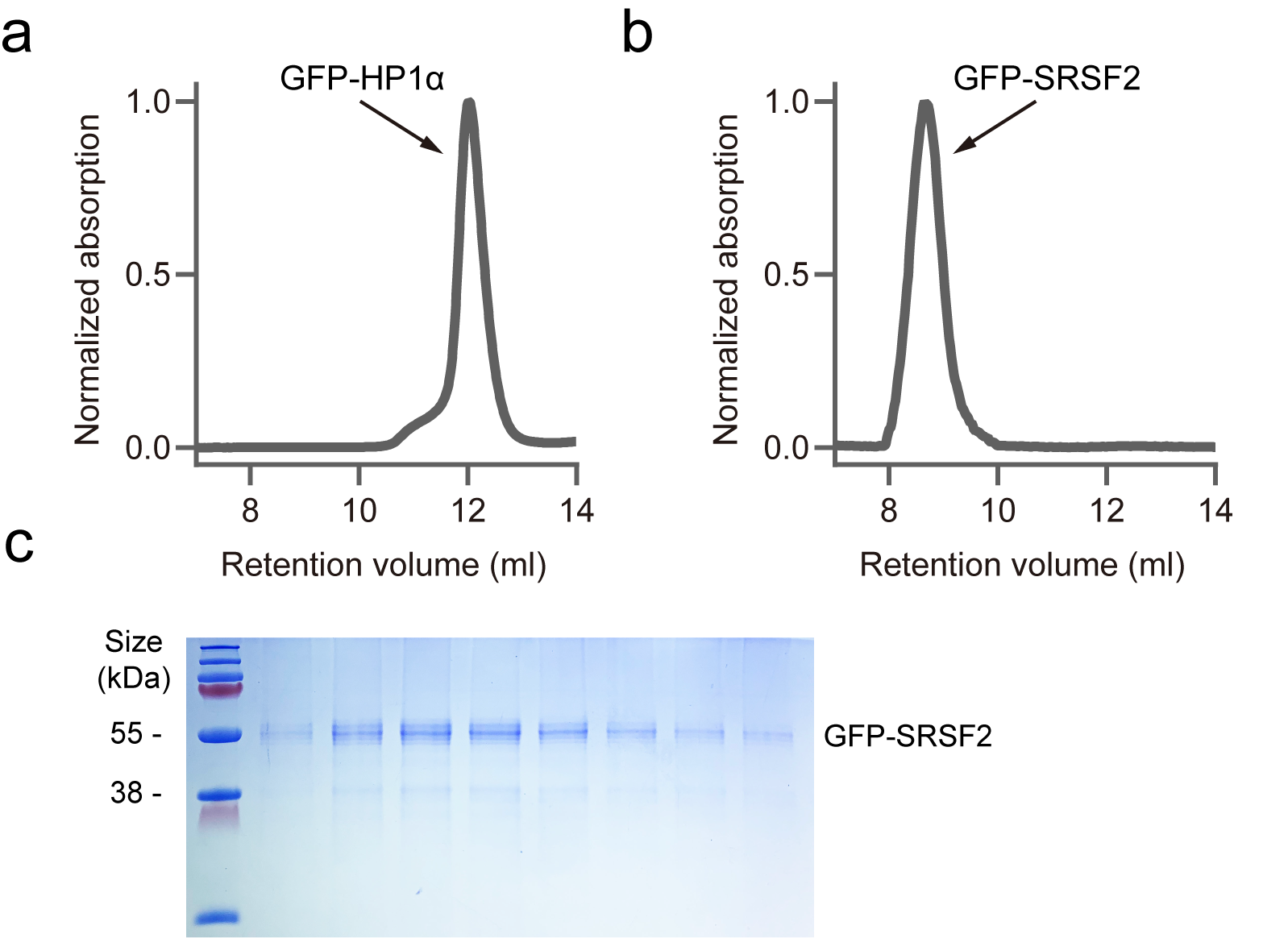


**Figure S1. Prokaryotic expression and purification of HP1α and SRSF2 protein. a, b** Gel-filtration chromatogram of the purified mEGFP-HP1α protein (**a**) and mEGFP-SRSF2 protein (**b**). **c** SDS-PAGE analysis of the mEGFP-SRSF2 protein in (**b**).


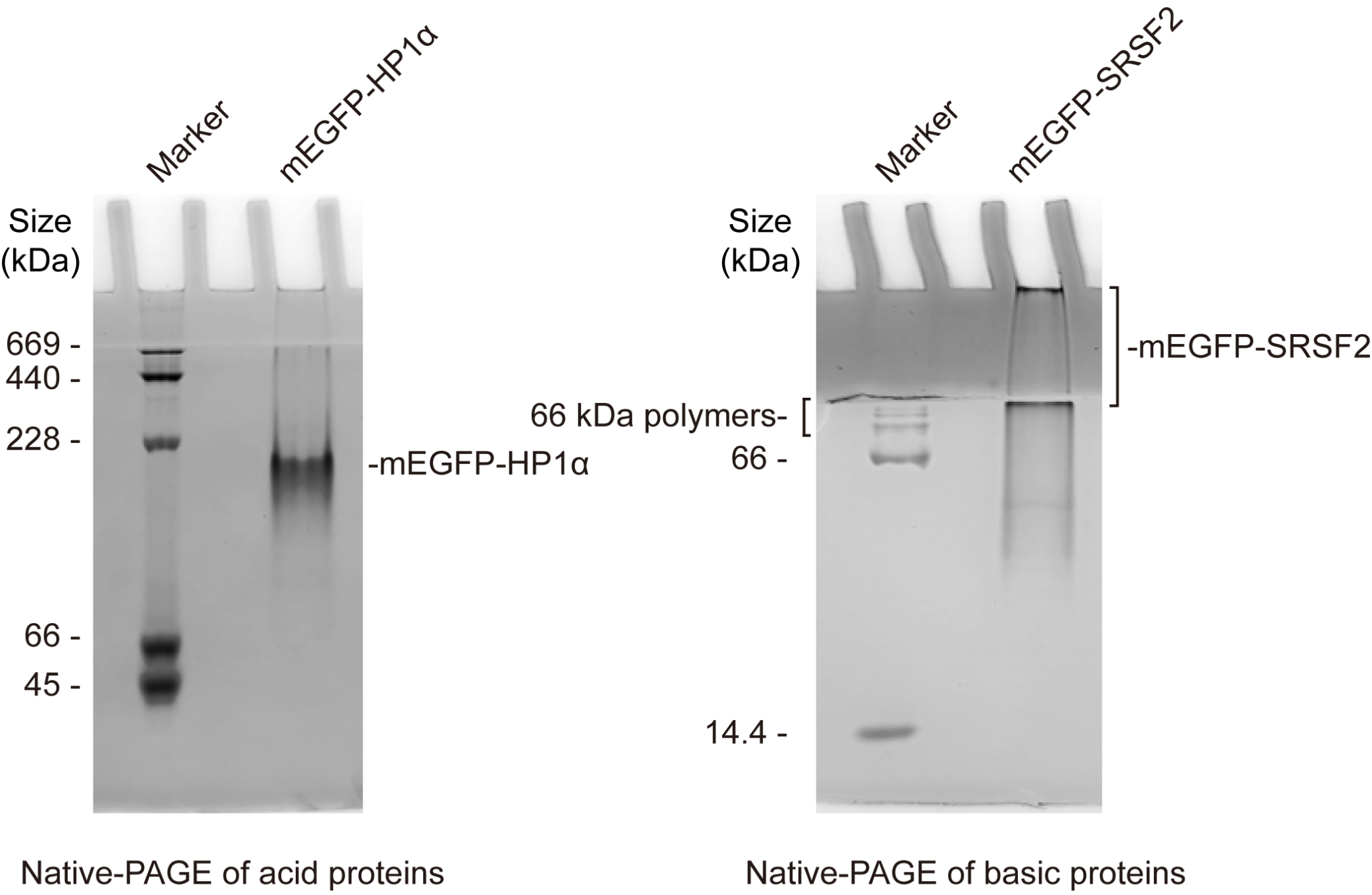


**Figure S2. Native-PAGE of purified mEGFP-HP1α and mEGFP-SRSF2 proteins.** 10 μg protein was loaded.

*
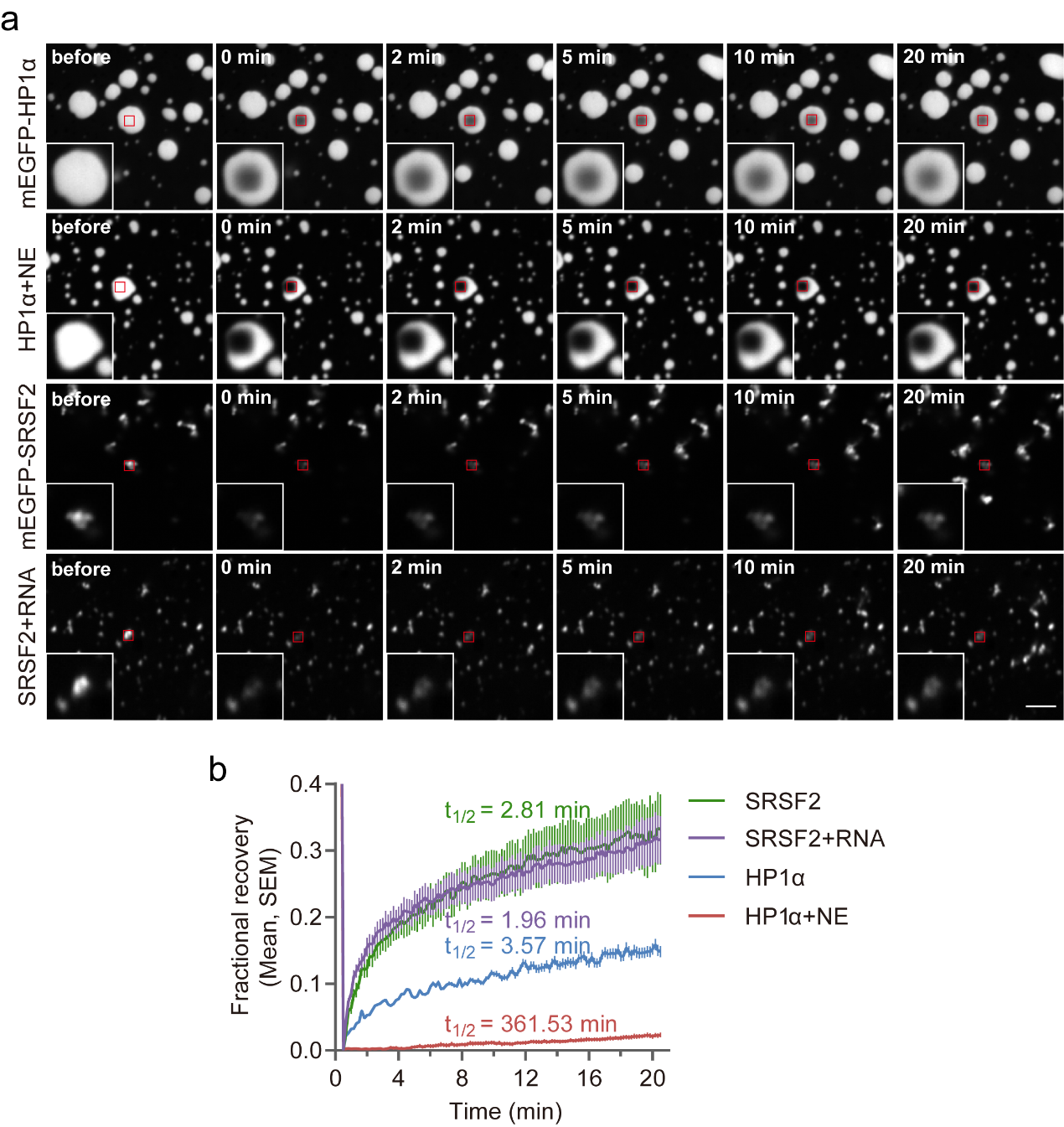
*

**Figure S3. Exploring the effect of nucleic acids on the mobility of SRSF2 and HP1α condensates. a** Representative images of mEGFP-HP1α and mEGFP-SRSF2 from the FRAP experiment. Scale bar, 5 μm. **b** Quantification of FRAP data. The bleaching event occurred at t = 10 s. N = 10 per group. Data are represented as mean ± SEM. NE, nuclear extracts.

**
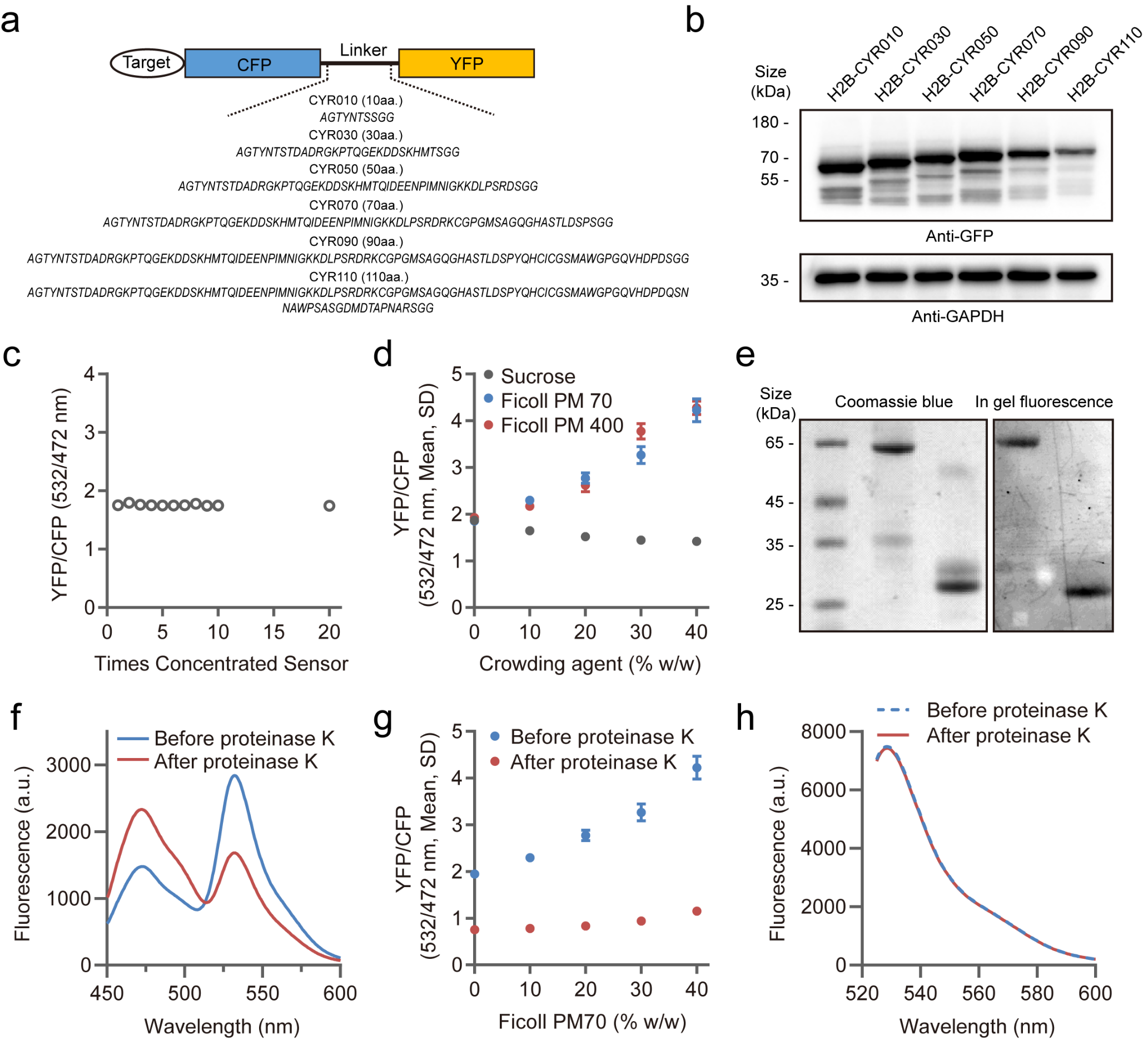
**

**Figure S4. Verification of the specificity of the CYR090 sensor. a** Schematic diagram of the optimized sensor to detect the degree of crowding containing random amino acid sequence linkers of different lengths. Prediction using the PSIPRED website (http://bioinf.cs.ucl.ac.uk/psipred/) confirmed that these linkers do not form a stable secondary structure, verifying their flexibility. **b** Western blot analysis of the H2B-fused sensors used to detect the degree of crowding shown in (**a**). **c** YFP/CFP emission ratio of the prokaryotically expressed and purified CYR090 sensor at different concentrations. **d** YFP/CFP emission ratio upon titration with sucrose, Ficoll PM 70, and Ficoll PM 400. N = 3 independent experiments. Data are represented as mean ± SD. **e** Proteinase K was used to cleave the flexible linker region of the CYR090 sensor to verify the specificity of the sensor. SDS-PAGE and in-gel fluorescence analysis of the cleavage reaction. **f** Fluorescence emission spectra after CFP excitation at 435 nm before and after proteinase K treatment. **g** YFP/CFP emission ratio upon titration with Ficoll PM 70 before and after proteinase K treatment. N = 3 independent experiments. Data are represented as mean ± SD. **h** Fluorescence emission spectra after YFP excitation at 515 nm before and after proteinase K treatment.


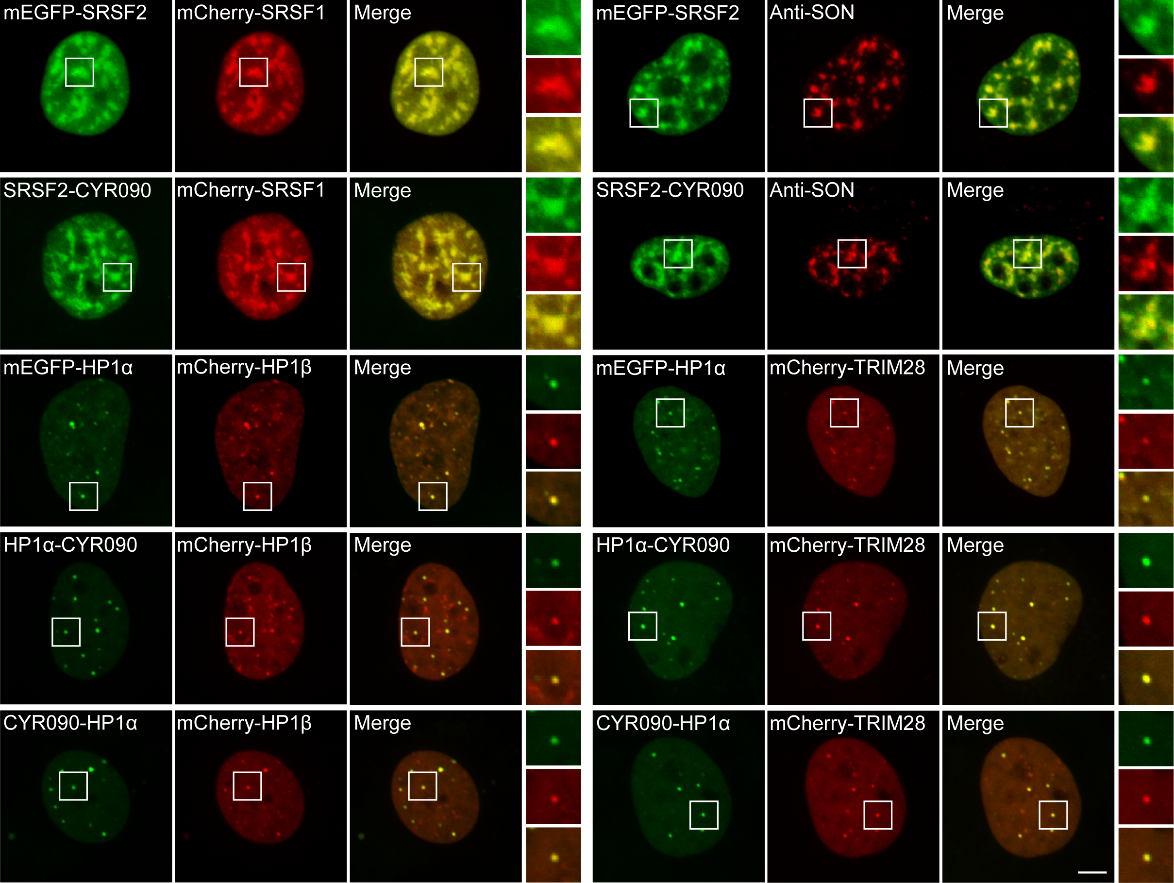


**Figure S5. Detecting the effect of CYR090 sensor on the function of SRFS2 or HP1α by fluorescence co-localization observation.** Except for immunofluorescence staining of SON protein, all other proteins were directly observed by confocal microscopy in living cells after overexpression. Scale bar, 5 μm.


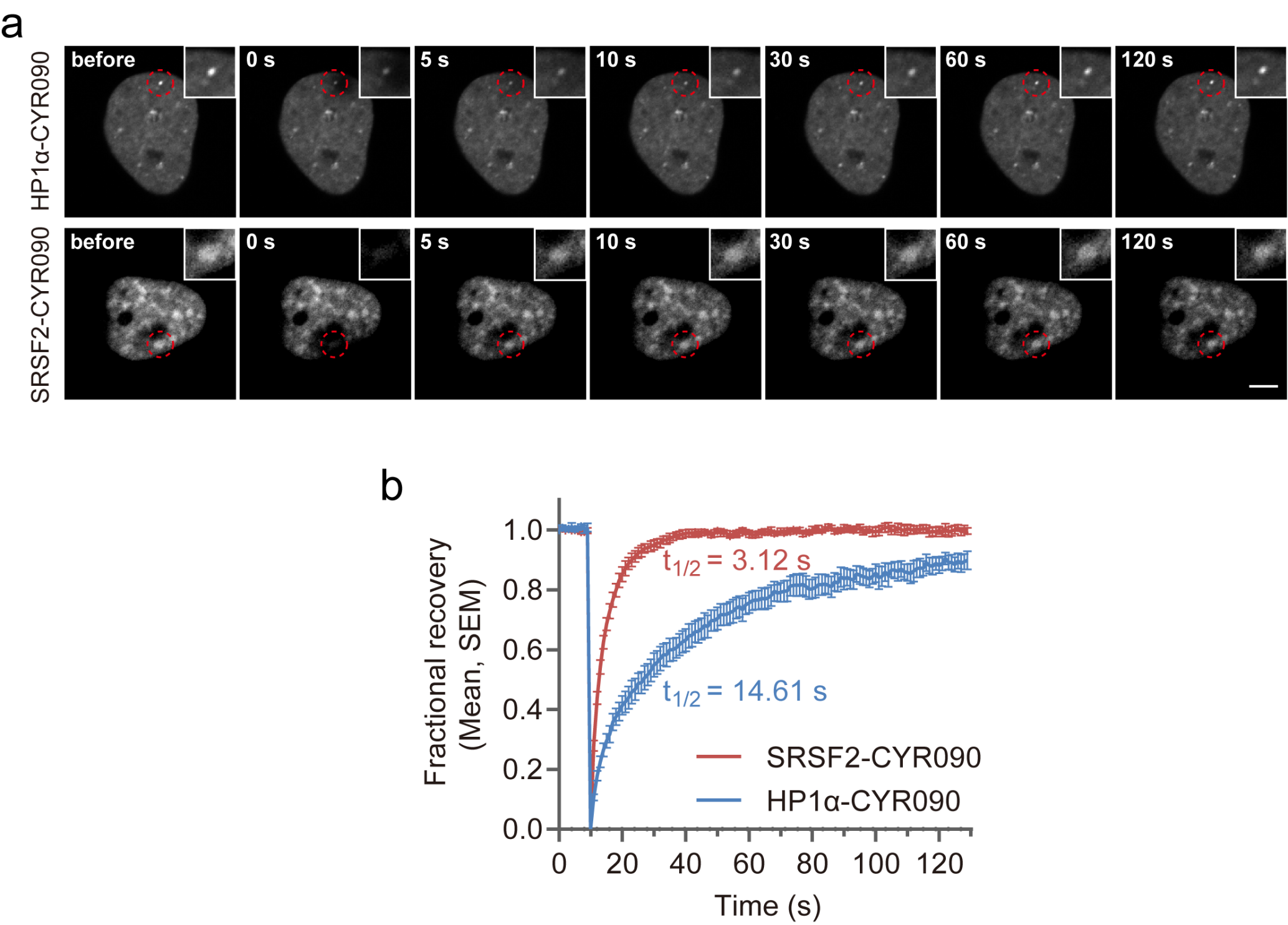


**Figure S6. Detecting the effect of CYR090 sensor on the mobility of SRFS2 or HP1α by FRAP assay. a** Representative images of HP1α-CYR090 and SRSF2-CYR090 from the FRAP experiment. Scale bar, 5 μm. **b** Quantification of FRAP data. The bleaching event occurred at t = 10 s. N = 8 cells per group. Data are represented as mean ± SEM.


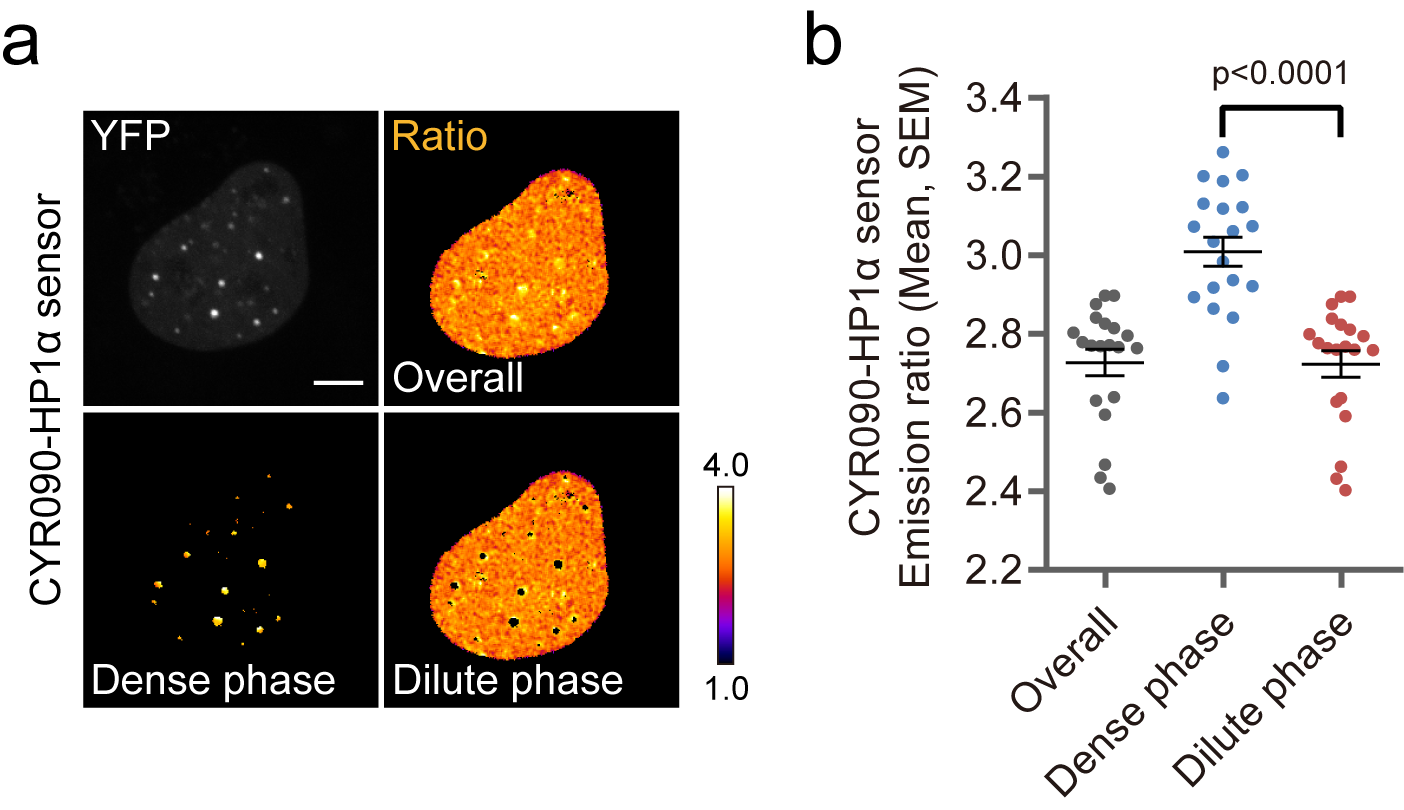


**Figure S7. Detecting the changes in crowding degree around HP1α with N-terminus fused CYR090 sensor. a** A representative image of CYR090-HP1α sensor, in which CYR090 sensor was fused to the N-terminus of HP1α, fluorescence intensity (YFP) and corresponding color-coded image of the overall and separate YFP/CFP emission ratio. Scale bar, 5 μm. **b** Comparison of YFP/CFP emission ratio of CYR090-HP1α sensor in different regions. N = 20 cells per group. Data are represented as mean ± SEM.


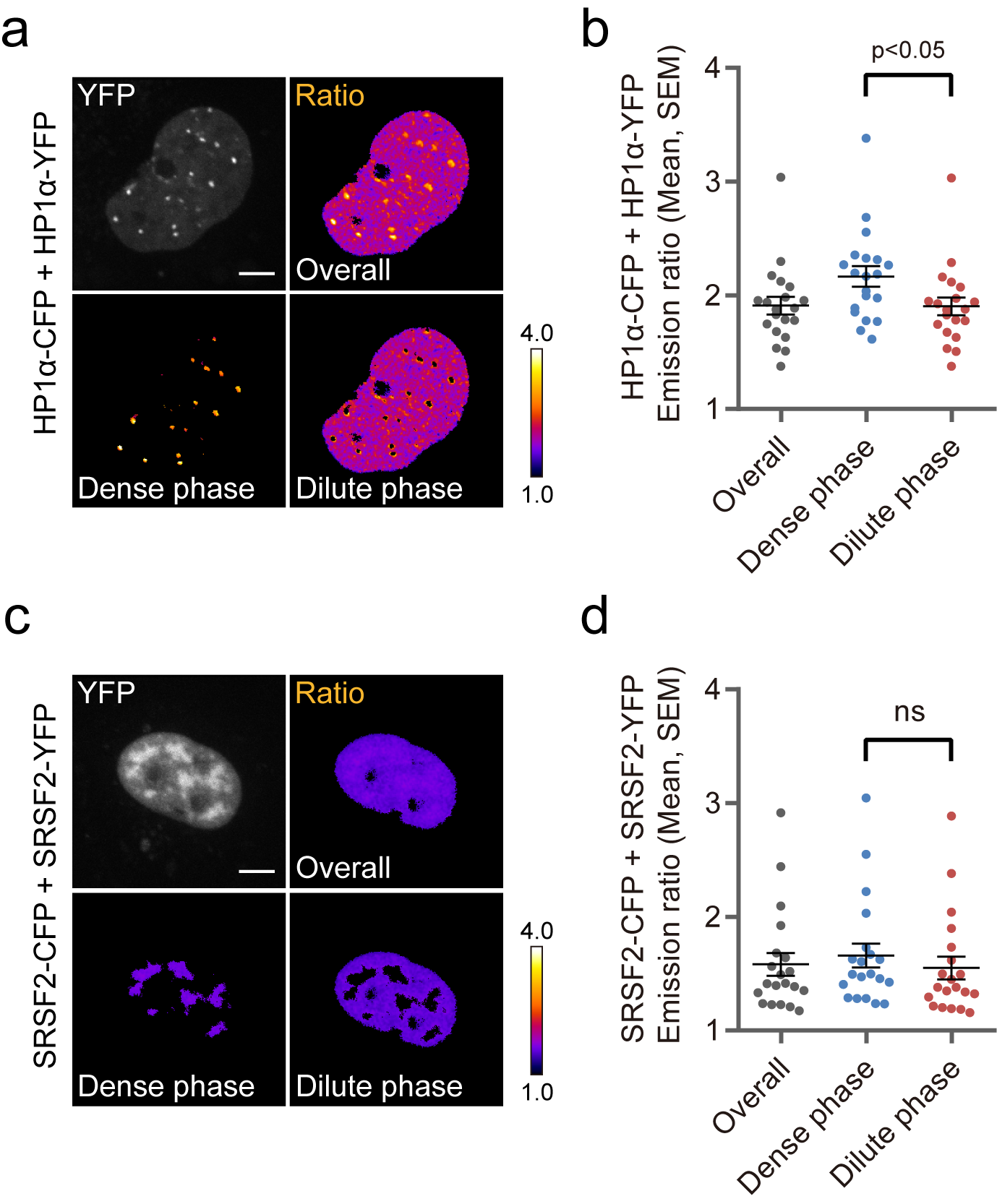


**Figure S8. Detecting the changes in crowding degree around HP1α and SRSF2 during phase separation by intermolecular FRET assay. a** A representative image of HP1α-YFP fluorescence intensity (YFP) and corresponding color-coded image of the overall and separate YFP/CFP emission ratio. Scale bar, 5 μm. **b** Comparison of YFP/CFP emission ratio in different regions of cells cotransfected HP1α-CFP and HP1α-YFP. N = 20 cells per group. Data are represented as mean ± SEM. **c** A representative image of SRSF2-YFP fluorescence intensity (YFP) and corresponding color-coded image of the overall and separate YFP/CFP emission ratio. Scale bar, 5 μm. **d** Comparison of YFP/CFP emission ratio in different regions of cells cotransfected SRSF2-CFP and SRSF2-YFP. N = 20 cells per group. Data are represented as mean ± SEM.


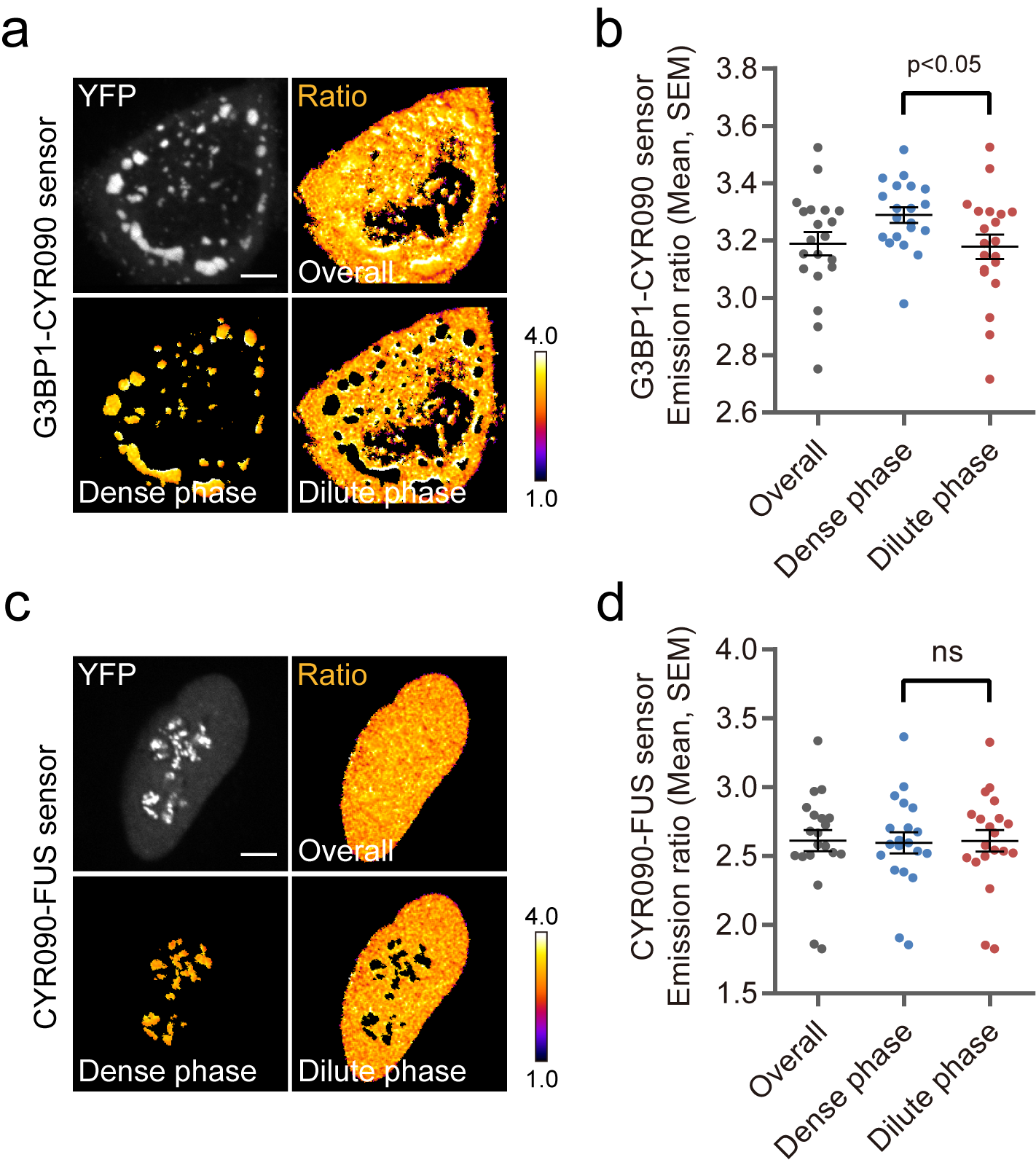


**Figure S9. Detecting the changes in crowding degree around G3BP1 and FUS during phase separation by CYR090 sensor. a** A representative image of G3BP1-CYR090 sensor fluorescence intensity (YFP) and corresponding color-coded image of the overall and separate YFP/CFP emission ratio. Cells were treated with 500 μM arsenite for one hour before observation. Scale bar, 5 μm. **b** Comparison of YFP/CFP emission ratio of G3BP1-CYR090 sensor in different regions. N = 20 cells per group. Data are represented as mean ± SEM. **c** A representative image of CYR090-FUS sensor fluorescence intensity (YFP) and corresponding color-coded image of the overall and separate YFP/CFP emission ratio. 5 μM AdOx was added to the cells 3 hours before transfection. Scale bar, 5 μm. **d** Comparison of YFP/CFP emission ratio of CYR090-FUS sensor in different regions. N = 20 cells per group. Data are represented as mean ± SEM.


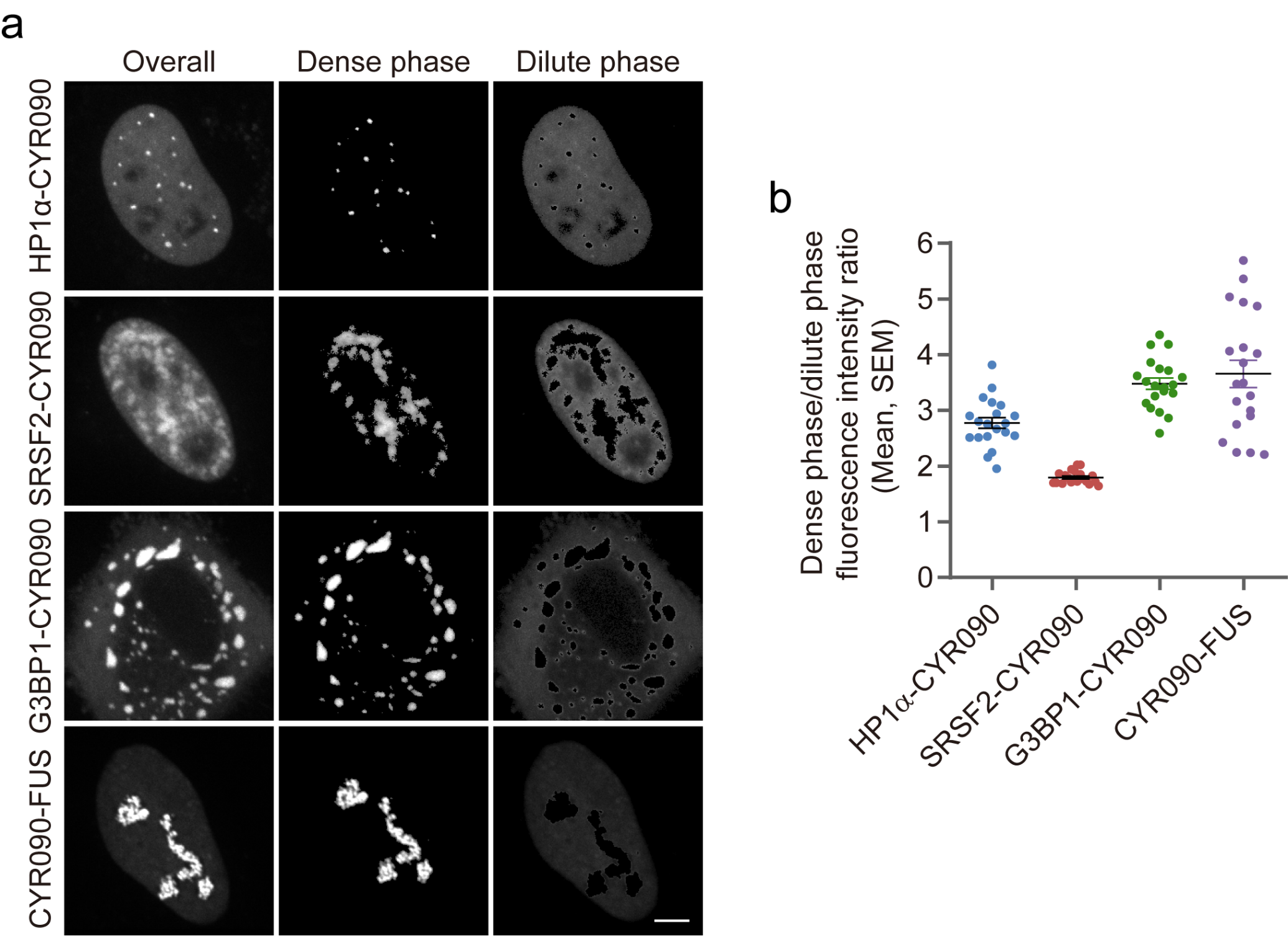


**Figure S10. Fluorescence intensity ratio of dense phase and dilute phase of CYR090 sensors fused to different target proteins. a** Representative images of CYR090 sensors fused with different target proteins. YFP fluorescence images were split into two parts according to where phase separation occurred. Scale bar, 5 μm. **b** Comparison of dense phase/dilute phase fluorescence intensity ratio of CYR090 sensors fused to different target proteins. N = 20 cells per group. Data are represented as mean ± SEM.


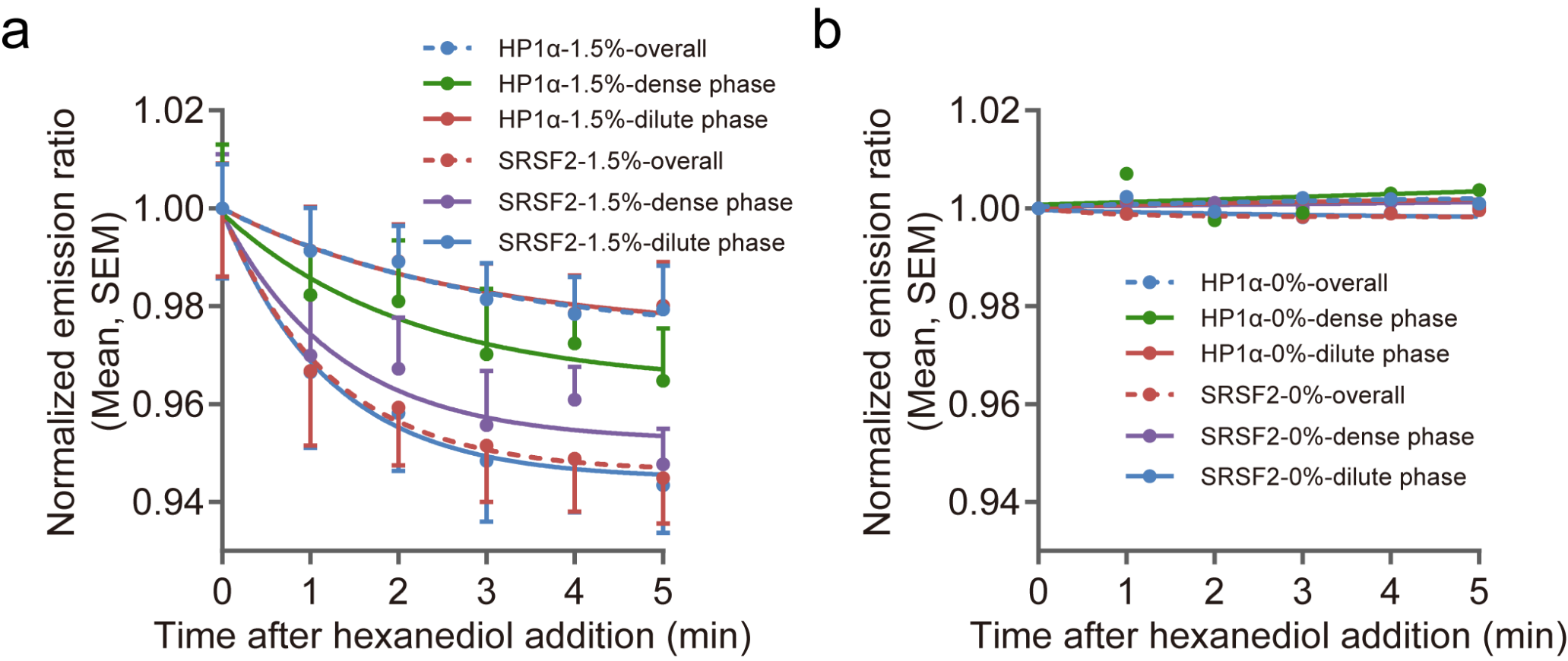


**Figure S11. Characteristics of intermolecular forces in different phase separation models. a** Statistical analysis of the influence of 1,6-hexanediol on the degree of HP1α and SRSF2 crowding using the HP1α-CYR090 sensor and SRSF2-CYR090 sensor; 1,6-hexanediol was added at 0 min. N = 10 cells per group. Data are represented as mean ± SEM. **b** Control group without 1,6-hexanediol. N = 10 cells per group. Data are represented as mean ± SEM. The emission ratios in (**a**) and (**b**) were normalized according to the YFP/CFP emission ratio at the beginning of the measurement.

**Original and uncropped films of Western blots**

Fig. 1g Anti-HP1α


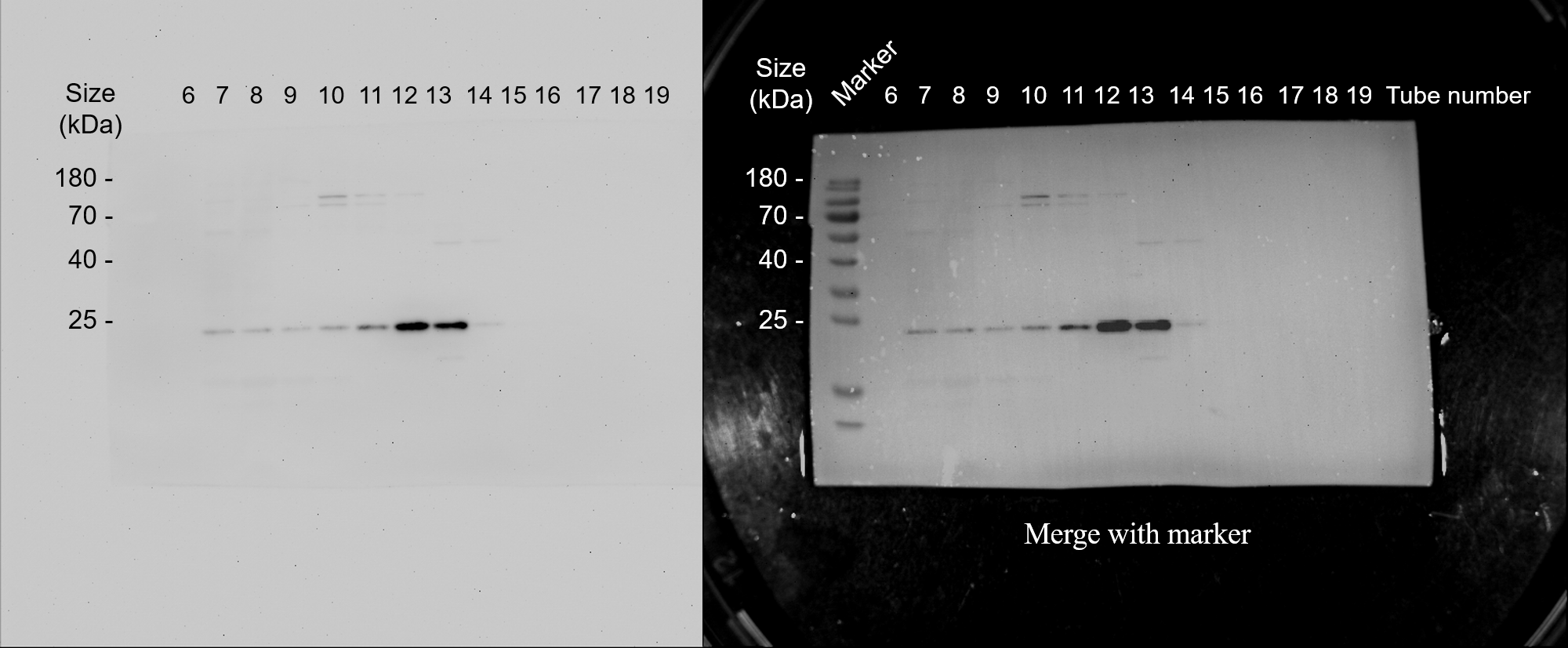


Fig. 1g Anti-SRSF2


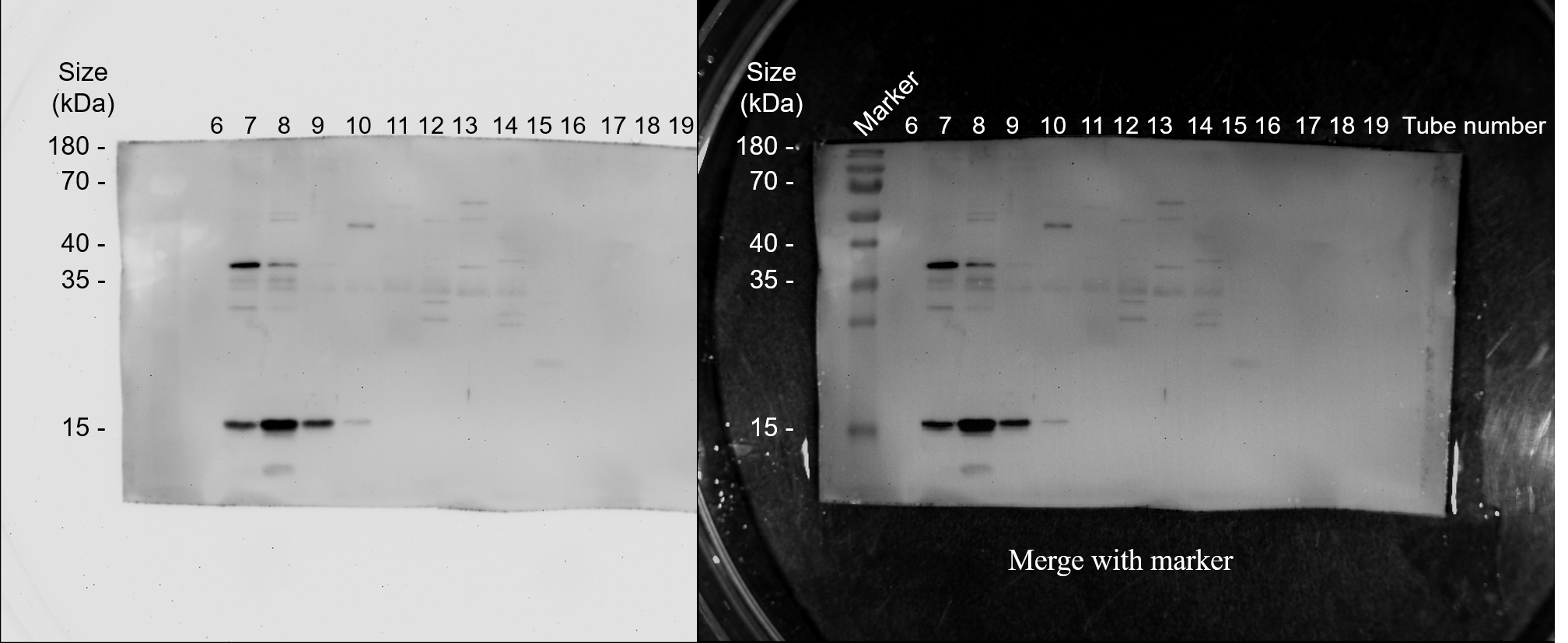


Supplementary Fig. S4b Anti-GFP


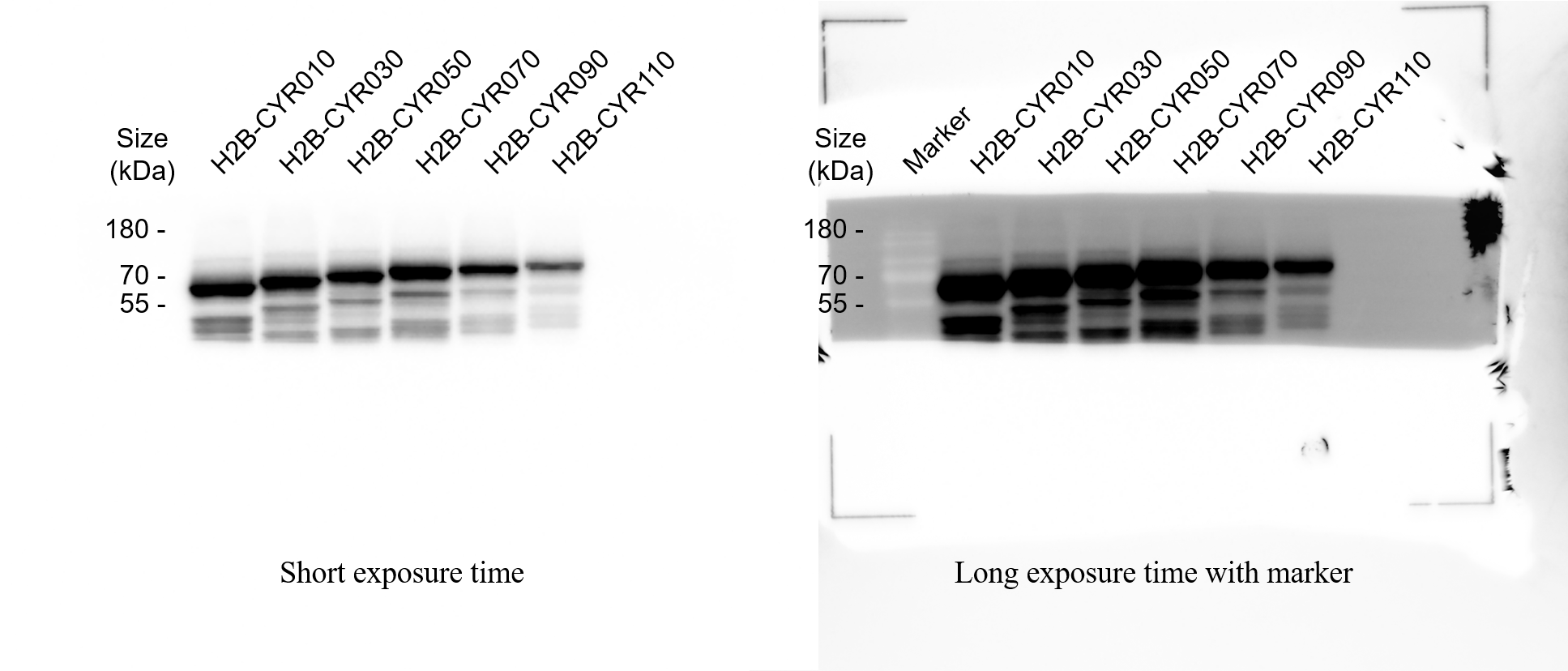


Supplementary Fig. S4b Anti-GAPDH


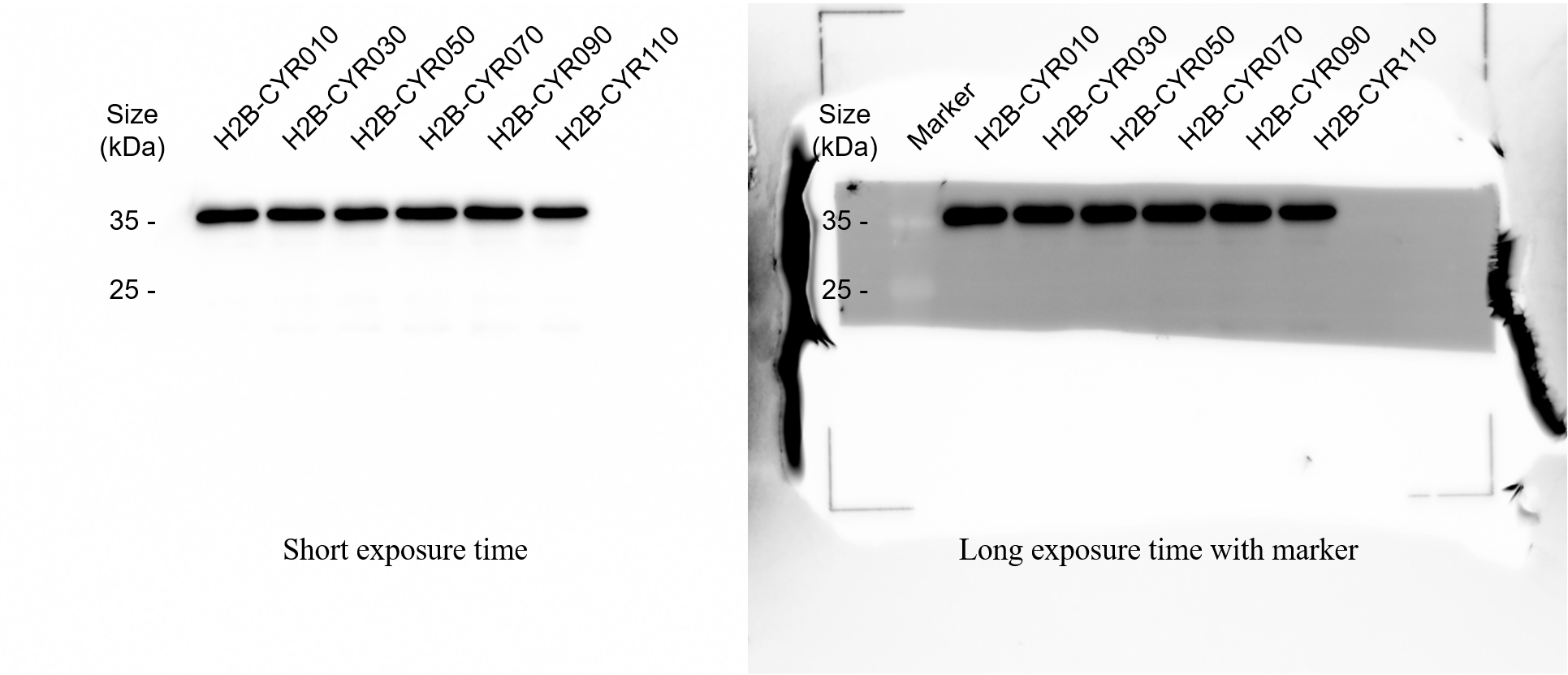


**Supplemental references**

1 Nguyen, A. W. & Daugherty, P. S. Evolutionary optimization of fluorescent proteins for intracellular FRET. *Nat. Biotechnol.* **23**, 355-360 (2005).

2 Zacharias, D. A., Violin, J. D., Newton, A. C. & Tsien, R. Y. Partitioning of lipid-modified monomeric GFPs into membrane microdomains of live cells. *Science* **296**, 913-916 (2002).

3 Klein, I. A. *et al.* Partitioning of cancer therapeutics in nuclear condensates. *Science* **368**, 1386-1392 (2020).

4 Boersma, A. J., Zuhorn, I. S. & Poolman, B. A sensor for quantification of macromolecular crowding in living cells. *Nat. Methods* **12**, 227-229, 221 p following 229 (2015).

5 Fuller, B. G. *et al.* Midzone activation of aurora B in anaphase produces an intracellular phosphorylation gradient. *Nature* **453**, 1132-1136 (2008).

6 Liu, D., Vader, G., Vromans, M. J., Lampson, M. A. & Lens, S. M. Sensing chromosome bi-orientation by spatial separation of aurora B kinase from kinetochore substrates. *Science* **323**, 1350-1353 (2009).

7 Trivedi, P. *et al.* The inner centromere is a biomolecular condensate scaffolded by the chromosomal passenger complex. *Nature Cell Biology* **21**, 1127-1137 (2019).

8 Ilik, I. A. *et al.* SON and SRRM2 are essential for nuclear speckle formation. *Elife* **9** (2020).

9 Wang, L. *et al.* Histone Modifications Regulate Chromatin Compartmentalization by Contributing to a Phase Separation Mechanism. *Mol. Cell* **76**, 646-659 e646 (2019).

10 Alberti, S., Gladfelter, A. & Mittag, T. Considerations and Challenges in Studying Liquid-Liquid Phase Separation and Biomolecular Condensates. *Cell* **176**, 419-434 (2019).

11 Elbaum-Garfinkle, S. Matter over mind: Liquid phase separation and neurodegeneration. *J. Biol. Chem.* **294**, 7160-7168 (2019).

12 Duster, R., Kaltheuner, I. H., Schmitz, M. & Geyer, M. 1,6-Hexanediol, commonly used to dissolve liquid-liquid phase separated condensates, directly impairs kinase and phosphatase activities. *J. Biol. Chem.* **296**, 100260 (2021).

13 Dundr, M. *et al.* In vivo kinetics of Cajal body components. *J. Cell Biol.* **164**, 831-842 (2004).

14 Phair, R. D. & Misteli, T. High mobility of proteins in the mammalian cell nucleus. *Nature* **404**, 604-609 (2000).

15 Weidtkamp-Peters, S. *et al.* Dynamics of component exchange at PML nuclear bodies. *J. Cell Sci.* **121**, 2731-2743 (2008).

16 Guo, Y. E. *et al.* Pol II phosphorylation regulates a switch between transcriptional and splicing condensates. *Nature* **572**, 543-548 (2019).

17 Larson, A. G. *et al.* Liquid droplet formation by HP1alpha suggests a role for phase separation in heterochromatin. *Nature* **547**, 236-240 (2017).

18 Strom, A. R. *et al.* Phase separation drives heterochromatin domain formation. *Nature* **547**, 241-245 (2017).

19 Jiang, S., Fagman, J. B., Chen, C., Alberti, S. & Liu, B. Protein phase separation and its role in tumorigenesis. *Elife* **9** (2020).

20 Zhang, H. *et al.* Liquid-liquid phase separation in biology: mechanisms, physiological functions and human diseases. *Sci. China Life Sci.* **63**, 953-985 (2020).

21 Bonnal, S. C., Lopez-Oreja, I. & Valcarcel, J. Roles and mechanisms of alternative splicing in cancer - implications for care. *Nat. Rev. Clin. Oncol.* **17**, 457-474 (2020).

22 Luo, C. *et al.* SRSF2 Regulates Alternative Splicing to Drive Hepatocellular Carcinoma Development. *Cancer Res.* **77**, 1168-1178 (2017).

23 Kim, E. *et al.* SRSF2 Mutations Contribute to Myelodysplasia by Mutant-Specific Effects on Exon Recognition. *Cancer Cell* **27**, 617-630 (2015).
